# Heredity Indexes for Estimating Heritability Using Known and Unknown Family Data Based on the Model of Polygenic Inheritance

**DOI:** 10.1155/2020/7243976

**Published:** 2020-03-26

**Authors:** Hui Liu

**Affiliations:** College of Medical Laboratory, Dalian Medical University, Dalian 116044, China

## Abstract

**Objective:**

To establish a model for estimating genetic risk using known and unknown family data.

**Methods:**

Four simulated datasets were generated for four paternal and maternal chromosomes. The simulated data for children were generated from the parental data according to the Mendelian law. The correlation coefficient between the children's and paternal data was calculated, and 2*R* was defined as the heredity index for continuous data (HIC). The simulated continuous data were transformed into binary data according to the gene accumulation threshold (incidence); the incidences of children in the parental no-disease group and the disease onset group were obtained; the correlation coefficient (*R*) was calculated as expected *R* (Re). The ratio of observed *R* (Ro) and Re was defined as the Heredity index for binary data (HIB).

**Results:**

Different actual pedigree data (lunula and holding a hammer in the right or left hand) were successfully used to verify the accuracy of the model. The genetic risk was estimated according to the incidence in a population using a lookup table.

**Conclusion:**

Our findings indicate the reliability of the model based on the fact that the multigene effect constitutes the normal distribution. Thus, this model can be used for comprehensive analysis of the influence of genetic and nongenetic factors on the genetic phenotype and to estimate genetic risk using known and unknown family data.

## 1. Introduction

Polygenic inheritance is a genetic trait controlled by multiple pairs of nonalleles. Multigene inheritance is more complicated than single-gene (Mendelian) inheritance. In polygenic heritable diseases, susceptibility is affected by both genetic and environmental factors, making research into the mechanism difficult [1–3]. Single-gene hereditary traits are qualitative in nature, and the variation between them is discontinuous. Polygenic hereditary phenotypes, such as height, body weight, blood pressure, and intelligence, are quantitative in nature, and the variation between them is continuous. The distribution of many traits forms a bell-shaped curve, which is similar to the distribution of traits determined by several genes. Each gene either enhances or weakens the trait and acts by external mechanisms that are not influenced by other genes. There are very few individuals at the two extremes of the distribution curve, while most are concentrated in the middle of the curve because each individual does not inherit many factors that act in the same direction. Environmental factors can enhance or weaken the final result, resulting in a normal distribution.

Many chronic diseases do not conform to the single-gene inheritance law (Mendelian inheritance) but follow a pattern that is closer to polygenic inheritance, with a threshold of separation between the susceptible and nonsusceptible individual [4]. There is a higher risk of developing diseases in the first-degree relatives (siblings and offspring) who carry 50% of the affected patient's genes. In contrast, the risk is much smaller in distant relatives who inherit only a few high susceptibility genes.

For polygenic genetic diseases, susceptibility is affected by both genetic and environmental factors [5–7]. Heritability describes the degree to which the variation in a particular trait, such as disease susceptibility, is influenced by genetics and is generally expressed as a percentage (%). If the susceptibility variability and morbidity of a disease are all determined by genetic factors, the heritability of the polygenic genetic disease is 100% although this case is rare. A general heritability of 50% or more indicates that genetic factors play an important role in determining susceptibility variability and morbidity, and environmental factors are less influential, that is, the heritability rate is high [8–10].

Heritability can be calculated using different methods. These calculations require close relative or even twin data, which are difficult to collect [11, 12]. Therefore, the data collection standard is broadened, and the accuracy of the data is difficult to guarantee. For most diseases, reliable data on close relatives are scarce, which limits both the application of the concept of heritability and in-depth studies on the disease.

In polygenic genetics, single-gene inheritance follows the Mendelian codominant model, that is, 50% of the characteristics of both parents is transmitted to their offspring. These features are suitable for establishing observational models of multigene genetic laws of inheritance from parents to children using data simulated for the pedigree. In this study, normally distributed data were simulated for four chromosomes from the father and the mother; the numerical values represented the enhancement or attenuation of the trait associated with each gene in the polygenic inheritance model; genetic traits of offspring were predicted according to the Mendelian law. A heritability analysis model was established without complex pedigree data, and it was also aimed to establish a presumption method of heredity with no family data or incomplete family data.

## 2. Methods

### 2.1. Theory of the Analytical Model

The basic assumptions of the analytical model are as follows: (1) the genetic characteristics of the research target are subject to polygenic inheritance with dominant expression. For a single gene, inheritance follows the Mendelian law; (2) the contribution of each gene to the trait is either strong, medium, or weak. Different genes play a role in a superimposed manner, and the combination of strong and weak genes is moderate, regardless of interaction or weight; (3) the polygenic hereditary phenotype belongs to quantitative traits, and the variation between the traits is continuous and normally distributed in a population; (4) the group genes are composed of known and unknown genes; therefore, the distribution of these genes is unknown; (5) there is a threshold that clearly separates the disease-affected from the nonaffected individuals [13]. If environmental factors are not taken into consideration, the gene accumulation threshold is set to medium, with medium or above (including medium) representing the disease onset group, and moderate-to-low representing the no-disease occurrence group. A threshold can be represented with incidence of a disease; (6) the increase in the onset threshold is the result of resistance factors; the role of these factors is also considered as the genetic characteristics of the research target.

### 2.2. Model Building

Four simulated data groups were generated for four maternal and paternal chromosomes. Each normally distributed simulated dataset (100 ± 30, *n* = 10,000) was established on the SPSS platform according to random distribution and designated as *A*, *B*, *C*, and *D*; the numerical values were set as the effect degree of genes. The sum of *A* and *B* represents the paternal effect, and the sum of *C* and *D* represents the maternal effect; thus, according to the Mendelian law, the effect in the children is represented by 1/4 (*A* + *C*) + 1/4 (*A* + *D*) + 1/4 (*B* + *C*) + 1/4 (*B* + *D*). Different thresholds of genes were generated for the paternal, maternal, and children's data; for instance, for a 25% threshold, the top 25% of samples was assigned a value of 1, while all others were assigned a value of 0. The continuous variables were transformed into binary variables using this design.

The corresponding simulated data were also established using the same method. Four simulated datasets were also generated for four chromosomes for two brothers. Each normally distributed simulated dataset (100 ± 30, *n* = 10,000) was established as described previously and also designated as *A*, *B*, *C*, and *D*. According to the Mendelian law, the sum of *A* and *B* represents the effect in brother 1, and the sum of 1/4 (*A* + *B*) + 1/4 (*A* + *C*) + 1/4 (*B* + *D*) + 1/4 (*C* + *D*) represents the effect in brother 2. The continuous variables were also transformed into binary variables as described previously.

### 2.3. Heredity Index for Continuous Data

Height and body weight are represented by continuous data. The simulated data (continuous data) were used to simulate polygenic inheritance and evaluate the relationship between the children and either the father or mother according to correlation analysis. The correlation coefficients (*R*) were 0.510 between the children and their fathers and mothers, respectively. Because nongenetic factors were not incorporated into the method used to generate the simulated data, the *R* was considered to reflect only the effects of genetic factors and considered as expected *R* (Re). The ratio of observed *R* (Ro) and Re was defined as the heredity index for continuous data (HIC), where HIC = Ro/0.5.

A HIC of less than 1.0 indicates a lesser role of inheritance, with smaller HIC values indicating a greater impact of nongenetic factors. A HIC of more than 1.0 indicates the possibility that only a few stronger genes play a role in inheritance.

### 2.4. Heredity Index for Binary Data

Carrying or not carrying a gene represents binary data. The continuous simulated data were transformed into binary data according to the different thresholds of genes in the populations of fathers, mothers, and children. The fathers (or mothers) were divided into a no-disease group and an onset group. The incidence of children in the parental no-disease group and the onset group was obtained, and the correlation coefficient (Spearman method) was calculated. *R* from special numbers is listed Table 1 as Re between incidence in children with disease and no-disease fathers for different nature incidence.

It was considered that the ratio of Ro and Re (from Table 1) was defined as the heredity index for binary data (HIB), where HIB = Ro/Re.

Ro is calculated with actual data; Re is the correlation coefficient under an incidence regardless of nongenetic factors and from Table 1. A HIB of more than 1.0 indicates that only a few stronger genes play a role in inheritance rather than reflecting the phenomenon of polygenic hereditary.

### 2.5. Actual Data for Continuous Variables

**Figure 1:**
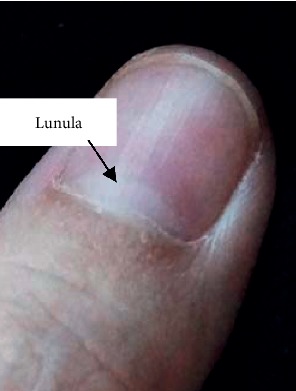
The lunula that is visible in some digits.

The lunula, which is the crescent-shaped white colored area at the base of a fingernail that is visible in some digits, is shown in Figure 1. This structure is formed by the visible part of the root of the nail and appears whiter than the rest of the nail because of the arrangement of the tissue in this area.

A total of 370 Chinese adults were randomly selected (186 male and 184 female) over the age of 18 years and their biological parents as participants in this study. The initial fingertip lunula count for each individual was collected through self-reporting or telephone interviews. The total lunula count for the 10 fingertips was considered as the continuous variable, and the correlation coefficient between children and their fathers was represented by HIC.

### 2.6. Actual Data for Binary Variables

Holding a hammer in the right or left hand was used as binary data. A total of 121 Chinese children (45 male and 76 female) and their parents (biological parents) were enrolled in the study. The data were obtained by self-reported surveys or telephone interviews. The genetic development of the gene intensity in the family was analyzed quantitatively using HIB. The proportion of the left-handed people is 15%, approximately [14].

## 3. Results

The siblings' data were used to replace the parents' data (the ratio of the nonincidence rate to the incidence rate on one side was calculated by dividing the other side into the no-disease and disease onset groups), and the HIB was calculated as shown in Table 2. The results were similar to those of the fathers and children for higher incidence rate.

The lunula counts in 10 fingertips of children in different father groups are shown in Table 3. The correlation coefficient was 0.549, implying HIC was 1.10.

The original data for holding a hammer in the right or left hand are shown in Table 4. Because the proportion of the left-handed people is 15% approximately [14], Re should be 0.272; the correlation coefficient was 0.293, implying that the HIB for the mode of holding a hammer was 1.10.

## 4. Discussion

In this study, a model was established for estimating genetic risk using known and unknown family data. HIC and HIB values were established based on the genetic coincidence rate. The model was based on the fact that the multigene effect constitutes the normal distribution of the effects of genes (strong or weak) in the population, and its theoretical basis is solid and reliable.

In terms of diseases, especially tumors or chronic diseases, their occurrence is greatly affected by nongenetic factors such as aging [15–17]. Furthermore, the incidence is low and family data are difficult to obtain [18]. Therefore, either of the parents was used to establish the model. When the siblings' data were used to replace the parents' data, the results should be similar to those of the fathers and children. It should be noted that when data for both parents were obtained, the father' data were suggested to be used although data from the mother or siblings could be used to replace missing data; when data for more siblings were obtained, the eldest sibling's data were suggested to be used.

In the calculations using actual data, HIC and HIB provided a reliable reflection of typical genetic phenomena (Tables 3 and 4). It should be pointed out that to simplify the calculation, the HIC or HIB value was not exactly equal to the actual heredity values and were therefore designated as the heredity index, which can be used for comprehensive analysis of the influence of genetic and nongenetic factors on the genetic phenotype.

The model established in this study can be used to estimate genetic risk according to prevalence in a population rather than that in the first-degree relatives. The incidence in each group can be obtained from Tables 1 and 2. For instance, if the disease prevalence is 1% in a population, the father or mother with disease implies a 17% incidence for their child; the sibling with disease implies a 34% incidence for their brothers and sisters. In contrast, the absence of disease in the father, mother, or first-degree relatives implies a maximum incidence of 0.8% for their child without consideration of nongenetic factors.

In conclusion, this model for estimating genetic risk based on the normal distribution of the multigene effect is reliable. Although the HIB or HIC value is not exactly equal to the actual heredity, this index provides a comprehensive analysis of the influence of genetic and nongenetic factors on the genetic phenotype and can be used to estimate genetic risk using known and unknown family data.

## Figures and Tables

**Table 1:** Relationship of nature incidence with the correlation coefficient between incidence in children with disease and no-disease fathers.

Nature incidence	Group for father	Incidence in children	Re
0.5	Onset	0.673	0.345
	No-disease	0.327	
0.25	Onset	0.501	0.335
	No-disease	0.166	
0.125	Onset	0.363	0.272
	No-disease	0.091	
0.0625	Onset	0.278	0.230
	No-disease	0.048	
0.010	Onset	0.170	0.162
	No-disease	0.008	

Re: correlation coefficients expected.

**Table 2:** Coincidence rate from siblings' data for different nature incidence.

Nature incidence	Group for father	Incidence in children	Re
0.5	Onset	0.707	0.413
	No-disease	0.293	
0.25	Onset	0.554	0.405
	No-disease	0.149	
0.125	Onset	0.298	0.303
	No-disease	0.067	
0.0625	Onset	0.392	0.351
	No-disease	0.041	
0.010	Onset	0.340	0.333
	No-disease	0.007	

Re: correlation coefficients expected.

**Table 3:** The lunula count in ten fingertips of children in different father groups.

Father group	Children (mean)
0	3.17
1	1.67
2	3.00
3	3.83
4	4.23
5	5.53
6	6.03
7	6.38
8	6.15
9	7.89
10	7.86
HIC	1.10

**Table 4:** Heredity index for binary data (HIB) for holding a hammer in the right or left hand.

Father groups	Total number	Children with left hammer holding (%)	HIB
Left hand	20	65.0	1.10
Right hand	101	27.7	

## Data Availability

The data used to support the findings of this study are available from the corresponding author upon request.
